# ﻿A new species of *Diploderma* Hallowell, 1861 (Squamata, Agamidae) discovered in the upper Dadu River valley of the Hengduan Mountains, Sichuan, China

**DOI:** 10.3897/zookeys.1251.153705

**Published:** 2025-09-03

**Authors:** Fengjing Liu, Yayong Wu, Jindong Zhang, Guang Yang, Shuo Liu, Xue Chen, Jiang Chang, Qiang Xie, Bo Cai

**Affiliations:** 1 Jiangsu Key Laboratory for the Biodiversity Conservation and Sustainable Utilization in the Middle and Lower Reaches of the Yangtze River Basin, College of Life Sciences, Nanjing Normal University, Nanjing 210023, China; 2 China-Croatia Belt and Road Joint Laboratory on Biodiversity and Ecosystem Services, Chengdu Institute of Biology, Chinese Academy of Sciences, Chengdu 610213, China; 3 Department of Agriculture, Forestry and Food Engineering, Yibin University, Yibin 644000, China; 4 College of Life Science, China West Normal University, Nanchong 637009, China; 5 Kunming Natural History Museum of Zoology, Kunming Institute of Zoology, Chinese Academy of Sciences, Kunming 650223, China; 6 Yunnan Key Laboratory of Biodiversity Information, Kunming Institute of Zoology, Chinese Academy of Sciences, Kunming 650201, China; 7 Sichuan Forestry Survey, Design Research Institute Co., Ltd, Chengdu 610084, China; 8 State Key Laboratory of Environmental Criteria and Risk Assessment, Chinese Research Academy of Environmental Sciences, Beijing 100012, China; 9 Sichuan Academy of Eco-Environmental Sciences, Chengdu 610041, China

**Keywords:** Agamidae, Barkam City, dry valley, Eastern Tibetan Plateau, Jinchuan County, lizard, ND2, taxonomy

## Abstract

A new species of the genus *Diploderma* is described from the upper Dadu River Valley in the Hengduan mountains of Sichuan Province, China. Phylogenetic analyses based on the mitochondrial *ND2* gene revealed that the new species, *Diploderma bifluviale***sp. nov.**, forms a distinct lineage within the *Diploderma flaviceps* group with an uncorrected genetic distance of ≥3.88%. Morphologically, the new species differs from its congeners by a combination of characters, including a shorter snout-vent length (SVL 62.51–72.55 mm), a shorter tail (TAL/SVL ratio 1.37–1.56), concealed tympanum, absence of a gular spot, and lemon-chiffon dorsolateral stripes with serrated edges in males. Additionally, *Diploderma bifluviale***sp. nov.** exhibits unique coloration traits such as a wheat-colored tongue and the fourth toe with claw reaching either the tympanum or the area between shoulder and tympanum when hind limbs adpressed forward. The species inhabits semi-arid shrublands in warm-dry valleys at elevations of 2,187–2,525 m – a habitat that is distinct from those of its closest relatives. This discovery highlights the understudied biodiversity of the upper Dadu River. *Diploderma bifluviale***sp. nov.** represents the 49^th^ species of the genus and expands our understanding of morphological and ecological diversity within the *D. flaviceps* group.

## ﻿Introduction

The genus *Diploderma*, belonging to the subfamily Draconinae (Squamata, Agamidae), is distributed across East Asia and the northern part of the Indochinese Peninsula (Wang et al. 2019a). To date, 46 species have been recognized within the genus *Diploderma*, making it one of the most species-rich genera among reptiles in China (Cai 2025). These lizards are characterized by their diverse morphology, color patterns, and ecological niches (Wang et al. 2024). Most species inhabit dry valley regions in the Hengduan mountains, exhibiting remarkable endemism and adaptability across a wide altitudinal range, particularly in the valleys of rivers such as the Jinsha, Yalong, Nu, and Lancang (Cai et al. 2024; Liu et al. 2024a).

**Figure 1.**
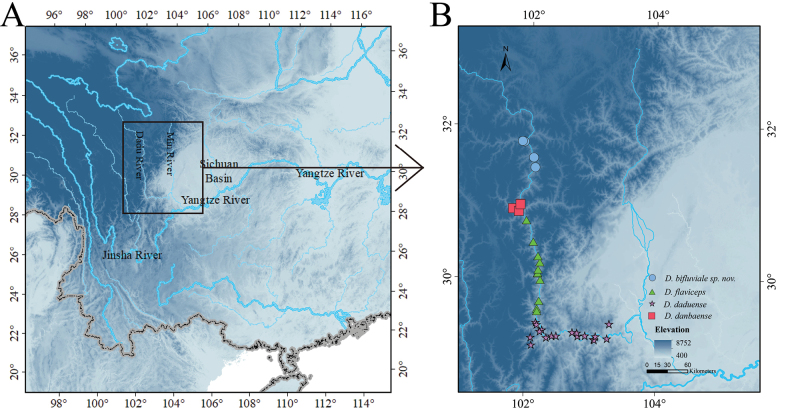
Map showing the type localities of *Diploderma bifluviale* sp. nov. (blue dot), *D. danbaense* (red square), *D. flaviceps* (green triangle), and *D. daduense* (purple five-pointed star), (B) in the Dadu River Valley, south-western China (A).

The Dadu River, a significant tributary of the Yangtze River, flows through the Hengduan Mountains and extends more than 1,062 kilometers in length. It traverses diverse landscapes from its origins in the highlands of Qinghai Province to its confluence with the Min River in Sichuan Province (Feng 2019). The river valley is characterized by rugged terrain and complex ecological systems. This unique environment has fostered endemic species of *Diploderma* (Fig. 1): *Diploderma daduense* (Cai, Liu & Chang., 2024), *Diploderma danbaense* Liu, Hou, Wang, Ananjeva & Orlov, 2023, and *Diploderma flaviceps* (Barbour & Dunn, 1919) (Cai et al. 2024). Although extensive research has been conducted on the middle and lower reaches of the Dadu River, the upper reaches have received relatively less attention until recently.

Since 2018, our team has conducted numerous surveys in the upper reaches of the Dadu River. During these surveys, we encountered a lizard species that exhibited distinct characteristics not previously observed among known *Diploderma* species in the Dadu River valley (Fig. 1). Through molecular biological analyses and morphological studies, we confirmed that this newly discovered lizard represents a previously unrecognized species within the genus *Diploderma*.

## ﻿Materials and methods

### ﻿Sampling

Field surveys in Sichuan Province were conducted from August 2018 by BC, from May 2023 by BC and FL, and from July and August by YW. Specimens were collected from 10:00 to 17:00 hr from the upper reaches of the Dadu River near Shuangjiangkou in Barkam City and Jinchuan County, Sichuan Province (Fig. 1). Live animals were photographed to document their color patterns before anesthesia and euthanasia. Euthanasia was performed through the intracelomic injection of 250 mg/kg of 1% MS-222 (Chengdu, China) for anesthesia, followed by intracelomic injection of 75% alcohol (Chengdu, China). Genetic tissues from livers were stored in 99% ethanol, and specimens were preserved in 75% ethanol. All newly collected specimens were deposited at the Museum of Herpetology, Chengdu Institute of Biology, Chinese Academy of Sciences (CIB).

### ﻿Morphological analysis

Museum abbreviations: The Museum of Herpetology, Chengdu Institute of Biology, Chinese Academy of Sciences (**CIB**); the Museum of Yibin Key Laboratory of Animal Diversity and Ecological Conservation, Yibin University (**YBU**); and Kunming Natural History Museum of Zoology, Kunming Institute of Zoology, Chinese Academy of Sciences (**KIZ**). Morphological data of recognized *Diploderma* species were obtained from the examination of museum specimens (Table 1), including the literature by Zhao et al. (1999), Liu et al. (2023), and Cai et al. (2024).

**Table 1.** GenBank accession numbers for the sequences used in this study.

	Species	Voucher	Locality	Accession no.	References
1	*Diploderma bifluviale* sp. nov.	20180822	Barkam, Sichuan, China	PV833282	This study
		CIB-CB23JC02	Barkam, Sichuan, China	PV833283	This study
		CIB-CB23JC03	Barkam, Sichuan, China	PV833284	This study
		CIB-CB23JC04	Barkam, Sichuan, China	PV833285	This study
		CIB-CB23JC05	Barkam, Sichuan, China	PV833286	This study
		CIB-CB23JC06	Barkam, Sichuan, China	PV833287	This study
		CIB-CB23JC07	Barkam, Sichuan, China	PV833288	This study
		CIB-CB23JC08	Barkam, Sichuan, China	PV833289	This study
		CIB-CB23JC19	Barkam, Sichuan, China	PV833292	This study
		CIB-CB23JC20	Barkam, Sichuan, China	PV833293	This study
		YBU23081/GP10410	Jinchuan, Sichuan, China	PV833294	This study
2	*D. angustelinea* Wang, Ren, Wu, Che & Siler, 2020	KIZ029704	Muli, Sichuan, China	MT577930	Wang et al. 2020
		KIZ029705	Muli, Sichuan, China	MT577924	Wang et al. 2020
3	*D. aorun* Wang, Jiang, Zheng, Xie, Che & Siler, 2020	KIZ032733	Benzilan, Yunnan, China	MT577938	Wang et al. 2020
		KIZ032734	Benzilan, Yunnan, China	MT577939	Wang et al. 2020
4	*D. batangense* (Li, Deng, Wu & Wang, 2001)	KIZ09404	Zhubalong, Tibet, China	MK001412	Wang et al. 2019a
		KIZ019276	Batang, Sichuan, China	MK001413	Wang et al. 2019a
		KIZ019277	Batang, Sichuan, China	MW133359	Che et al. 2021
5	*D. brevicauda* (Manthey, Denzer, Hou & Wang, 2012)	KIZ044305	Lijiang, Yunnan, China	MW506021	Wang et al. 2021
		KIZ044306	Lijiang, Yunnan, China	MW506022	Wang et al. 2021
6	*D. bowoense* Wang, Gao, Wu, Siler & Che, 2021	KIZ044757	Muli, Sichuan, China	MW506020	Wang et al. 2021
		KIZ044758	Muli, Sichuan, China	MW506019	Wang et al. 2021
7	*D. brevipes* (Gressitt, 1936)	NMNS19607	Taiwan, China	MK001429	Wang et al. 2019a
		NMNS19608	Taiwan, China	MK001430	Wang et al. 2019a
8	*D. chapaense* (Bourret, 1937)	KIZ034923	Lvchun, Yunnan, China	MG214263	Wang et al. 2018
		ZMMUNAP-01911	Chapa, Vietnam	MG214262	Wang et al. 2018
9	*D. danbaense* Liu, Hou, Wang, Ananjeva & Orlov, 2023	KIZ2022048	Danba, Sichuan, China	OQ378180	Liu et al. 2023
		KIZ2022049	Danba, Sichuan, China	OQ378181	Liu et al. 2023
		KIZ2022050	Danba, Sichuan, China	OQ378182	Liu et al. 2023
		CIB-CB23JC17	Danba, Sichuan, China	PV833290	This study
		CIB-CB23JC18	Danba, Sichuan, China	PV833291	This study
10	*D. daduense* (Cai, Liu & Chang., 2024)	CIB-CB2021227	Hanyuan, Sichuan, China	PP539949	Cai et al. 2024
		CIB-CB2021228	Hanyuan, Sichuan, China	PP539950	Cai et al. 2024
		CIB-CB2021229	Hanyuan, Sichuan, China	PP539951	Cai et al. 2024
		CIB-CB2021239	Jinkouhe, Sichuan, China	PP539955	Cai et al. 2024
		CIB-CB2021246	Shimian, Sichuan, China	PP539959	Cai et al. 2024
		CIB-CB2021249	Shimian, Sichuan, China	PP539962	Cai et al. 2024
		CIB-CB2021252	Jinkouhe, Sichuan, China	PP539963	Cai et al. 2024
11	*D. daochengense* Cai, Zhang, Li, Du, Xie, Hou, Zhou & Jiang, 2022	20210905	Muli, Sichuan, China	OP595620	Cai et al. 2022
		DC001	Daocheng, Sichuan, China	OP595621	Cai et al. 2022
12	*D. donglangense* Liu, Hou, Ananjeva & Rao, 2023	KIZ2022057	Muli, Sichuan, China	OQ378185	Liu et al. 2023
		KIZ2022058	Muli, Sichuan, China	OQ378186	Liu et al. 2023
13	*D. drukdaypo* (Wang, Ren, Jiang, Zou, Wu, Che & Siler, 2019)	KIZ027627	Jinduo, Tibet, China	MT577950	Wang et al. 2020
		KIZ027628	Zhuka, Tibet, China	MT577952	Wang et al. 2020
14	*D. dymondi* (Boulenger, 1906)	KIZ040639	Dongchuan, Yunnan, China	MK001422	Wang et al. 2019a
		KIZ040640	Dongchuan, Yunnan, China	MK001423	Wang et al. 2019a
15	*D. fasciatum* (Mertens, 1926)	SYS r002175	Wuming, Guangxi, China	OM055809	Wang et al. 2022
		KIZ040192	Daweishan, Yunnan, China	OM055800	Wang et al. 2022
16	*D. flaviceps* (Barbour & Dunn, 1919)	KIZ01851	Luding, Sichuan, China	MK001416	Wang et al. 2019a
		KIZ01852	Luding, Sichuan, China	MK001417	Wang et al. 2019a
17	*D. flavilabre* Wang, Che & Siler, 2020	KIZ032692	Baiyu,Sichuan, China	MT577916	Wang et al. 2020
		KIZ032694	Baiyu,Sichuan, China	MT577917	Wang et al. 2020
		KIZ032695	Baiyu,Sichuan, China	MT577918	Wang et al. 2020
18	*D. formosgulae* Wang, Gao, Wu, Dong, Shi, Qi, Siler & Che, 2021	KIZ044420	Deqin, Yunnan, China	MW506024	Wang et al. 2021
		KIZ044421	Deqin, Yunnan, China	MW506025	Wang et al. 2021
19	*D. iadinum* (Wang, Jiang, Siler & Che, 2016)	KIZ027697	Yunling, Yunnan, China	MT577956	Wang et al. 2020
		KIZ027702	Yunling, Yunnan, China	MT577957	Wang et al. 2020
20	*D. jiulongense* Liu, Hou, Ananjeva & Rao, 2023	KIZ2022086	Jiulong, Sichuan, China	OQ378190	Liu et al. 2023
		KIZ2022087	Jiulong, Sichuan, China	OQ378191	Liu et al. 2023
21	*D. kangdingense* Cai, Zhang, Li, Du, Xie, Hou, Zhou & Jiang, 2022	20210916	Kangding, Sichuan, China	OP595625	Cai et al. 2022
		20210917	Kangding, Sichuan, China	OP595626	Cai et al. 2022
22	*D. laeviventre* (Wang, Jiang, Siler & Che, 2016)	KIZ014037	Basu, Tibet, China	MK001407	Wang et al. 2019a
		KIZ027691	Basu, Tibet, China	MT577892	Wang et al. 2020
		KIZ027692	Basu, Tibet, China	MT577893	Wang et al. 2020
23	*D. limingense* Liu, Hou, Rao & Ananjeva, 2022	KIZ2022014	Yulong, Yunnan, China	OP428782	Liu et al. 2022
		KIZ2022015	Yulong, Yunnan, China	OP428783	Liu et al. 2022
		KIZ2022017	Yulong, Yunnan, China	OP428784	Liu et al. 2022
24	*D. luei* (Ota, Chen & Shang, 1998)	NMNS19604	Taiwan, China	MK001433	Wang et al. 2019a
		NMNS19605	Taiwan, China	MK001434	Wang et al. 2019a
25	*D. makii* (Ota, 1989)	NMNS19609	Taiwan, China	MK001431	Wang et al. 2019a
		NMNH19610	Taiwan, China	MK001432	Wang et al. 2019a
26	*D. menghaiense* Liu, Hou, Wang, Ananjeva & Rao, 2020	KIZ L0030	Menghai, Yunnan, China	MT598655	Liu et al. 2020
		KIZ L0031	Menghai, Yunnan, China	MT598656	Liu et al. 2020
27	*D. micangshanense* (Song, 1987)	KIZ032801	Shiyan, Hubei, China	MK578665	Wang et al. 2019b
		KIZ023231	Xixia, Henan, China	MK578664	Wang et al. 2019b
28	*D. panchi* Wang, Zheng, Xie, Che & Siler, 2020	KIZ032715	Yajiang, Sichuan, China	MT577946	Wang et al. 2020
		KIZ032716	Yajiang, Sichuan, China	MT577944	Wang et al. 2020
29	*D. panlong* Wang, Che & Siler, 2020	KIZ040137	Miansha, Sichuan, China	MT577906	Wang et al. 2020
		KIZ040138	Miansha, Sichuan, China	MT577907	Wang et al. 2020
30	*D. polygonatum* Hallowell, 1861	NMNS19598	Taiwan, China	MK001427	Wang et al. 2019a
		NMNS19599	Taiwan, China	MK001428	Wang et al. 2019a
31	*D. qilin* Wang, Ren, Che & Siler, 2020	KIZ028332	Deqin, Yunnan, China	MT577941	Wang et al. 2020
		KIZ028333	Deqin, Yunnan, China	MT577942	Wang et al. 2020
32	*D. shuoquense* Liu, Hou, Rao & Ananjeva, 2022	KIZ2022004	Xiangcheng, Sichuan, China	OP428773	Liu et al. 2022
		KIZ2022005	Xiangcheng, Sichuan, China	OP428774	Liu et al. 2022
33	*D. slowinskii* (Rao, Vindum, Ma, Fu & Wilkinson, 2017)	CAS214906	Gongshan, Yunnan, China	MK001405	Wang et al. 2019a
		CAS214954	Gongshan, Yunnan, China	MK001406	Wang et al. 2019a
		KIZ027543	Gongshan, Yunnan, China	MT577910	Wang et al. 2020
34	*D. splendidum* (Barbour & Dunn, 1919)	KIZ015973	Yichang, Hubei, China	MK001418	Wang et al. 2019a
		LSUMZ81212	Unknown	AF288230	McGuire and Heang 2001
35	*D. swild* Wang, Wu, Jiang, Chen, Miao, Siler & Che, 2019	KIZ034914	Panzhihua, Sichuan, China	MN266299	Wang et al. 2019c
		KIZ034894	Panzhihua, Sichuan, China	MN266300	Wang et al. 2019c
36	*D. swinhonis* (Günther, 1864)	NMNS19592	Taiwan, China	MK001419	Wang et al. 2019a
		NMNS19593	Taiwan, China	MK001420	Wang et al. 2019a
37	*D. tachengense* Liu, Hou, Ananjeva & Rao, 2023	KIZ2022028	Weixi, Yunnan, China	OQ378195	Liu et al. 2023
		KIZ2022027	Weixi, Yunnan, China	OQ378196	Liu et al. 2023
38	*D. varcoae* (Boulenger, 1918)	WK-JK 011	Yuxi, Yunnan, China	MT577903	Wang et al. 2020
		KIZ026132	Mengzi, Yunnan, China	MK001421	Wang et al. 2019a
39	*D. vela* (Wang, Jiang, Siler & Che, 2023)	KIZ019299	Quzika, Tibet, China	MK001414	Wang et al. 2019a
		KIZ034925	Quzika, Tibet, China	MK001415	Wang et al. 2019a
40	*D. xinlongense* Cai, Zhang, Li, Du, Xie, Hou, Zhou & Jiang, 2022	20210907	Xinlong, Sichuan, China	OP595613	Cai et al. 2022
		20210908	Xinlong, Sichuan, China	OP595614	Cai et al. 2022
41	*D. yangi* Wang, Zhang & Li, 2022	SWFU005410	Chayu, Tibet, China	OL449603	Wang et al. 2022
		SWFU005412	Chayu, Tibet, China	OL449604	Wang et al. 2022
		SWFU005414	Chayu, Tibet, China	OL449605	Wang et al. 2022
42	*D. yongshengense* (Wang, Ren, Jiang, Siler & Che, 2023)	KIZ2022009	Yongsheng, Yunnan, China	OP428777	Liu et al. 2022
		KIZ2022008	Yongsheng, Yunnan, China	OP428778	Liu et al. 2022
43	*D. yulongense* (Manthey, Denzer, Hou & Wang, 2012)	KIZ028291	Hutiaoxia, Yunnan, China	MT577921	Wang et al. 2020
		KIZ028292	Hutiaoxia, Yunnan, China	MT577922	Wang et al. 2020
44	*D. yunnanense* (Anderson, 1878)	CAS242271	Baoshan, Yunnan, China	MK001408	Wang et al. 2019a
		KIZ040193	Yingjiang, Yunnan, China	MK578658	Wang et al. 2019b
45	*D. zhaoermii* (Gao & Hou, 2002)	KIZ019564	Wenchuan, Sichuan, China	MK001425	Wang et al. 2019a
		KIZ019565	Wenchuan, Sichuan, China	MK001426	Wang et al. 2019a
46	*Pseudocalotes brevipes* (Werner, 1904)	MVZ224106	Vinh Phuc, Vietnam	AF128502	Macey et al. 2000
47	*Bronchocela cristatella* (Kuhl, 1820)	RMB8883	Unknown	KR053114	Grismer et al. 2015
48	*Laodracon carsticola* Luu, Nguyen, Le, Bonkowski & Ziegler, 2020	NUOL.R.2022.01	Khammuone, Laos	OR544068	Sitthivong et al. 2023

Measurements were conducted following the methods outlined by Zhao et al. (1999) and were taken to the nearest 1 mm using a steel ruler for snout-vent length and tail length, and to the nearest 0.1 mm using a digital caliper for other relatively short measurements. To minimize the influence of allometric effects, data from larvae and subadult individuals were excluded from morphological comparisons. A total of 11 specific measurements were recorded:

**SVL** Snout-vent length: from the tip of the snout to the anterior edge of the cloaca;

**TAL** Tail length: from the anterior edge of the cloaca to the tip of the tail;

**HW** Head width: measured between the widest points in the temporal region, anterior to the tympanum;

**HL** Head length: from the tip of the snout to the posterior angle of the jaw;

**HD** Head depth: at the temporal region of the head;

**SEL** Snout-eye length: from the tip of the snout to the anterior margin of the orbit;

**TNC** Length of the tallest nuchal crest: from the base to the apex of the tallest nuchal crest;

**FLL** Foreleg length: from the axilla to the tip of finger IV, excluding the claw, with the limb straightened;

**HLL** Hindleg length: from the groin to the tip of toe IV, excluding the claw, with the limb straightened;

**TRL** Trunk length: from the armpit to the groin;

**T4L** Toe IV length: from the tip of toe IV to the base between toes III and IV, excluding the claw.

The definitions of morphological characteristics and counting methods adhered primarily to Zhao et al. (1999) and Liu et al. (2023):

**SL** Supralabial scale count: labial scales from rostral to the corner of mouth;

**NSL** Nasal-supralabial scale rows: counted between the first supralabial and the nasal scale;

**IL** Infralabials: from the mental scale to the corner of the mouth;

**VN** Ventrals: counted in a straight line along the medial axis from the transverse gular fold to the anterior edge of the cloaca;

**GU** Gulars: counted in a straight line along the medial axis from and excluding the mental scale to the transverse gular fold;

**MD** Middorsal crest scales: counted longitudinally from the first nuchal crest scale to the scale above the cloaca;

**F4S** Finger IV subdigital lamellae: from the base between fingers III and IV to the tip of finger IV, excluding the claw;

**T4S** Toe IV subdigital lamellae: from the base between toes III and IV to the tip of toe IV, excluding the claw;

**RSBE** Radial stripes below the eyes: absent or present;

**GF** Gular fold state: absent or present;

**GP** Gular pouch state: absent or present;

**TS** Tympanum state: concealed by scales or exposed;

**SFNC** Skin fold under the nuchal crest: absent or present;

**NC** Nuchal crest state: strongly erected or not;

**SFDC** Skin fold under the dorsal crest: absent or present;

**SDS** Shape of dorsolateral stripes in males: smooth-edged or jagged;

**VSS** Ventral scale state: absent or present;

**HAF** Hindlimbs adpressed forward: measured as the area reached when hindlimbs are pressed forward;

**ST** Supratemporals: enlarged and modified temporal scales;

**SOR** Suborbital scale rows: longitudinal rows of scales between supralabials and inferior-most edge of orbit circle, excluding fine ciliary scales in the orbit.

Coloration descriptions utilized terminology and codes from the RGB (red, green, blue) color model (Cai et al. 2022). Data on coloration and ornamentation were gathered from live specimens both in unstressed and stressed states, including:

**GSC** Gular spot color;

**ILC** nner-lip coloration;

**CO** Coloration of the oral cavity: defined as the background coloration of the anterior roof and sides of the mouth, excluding the posterior palate and deep throat;

**CTG** Coloration of the tongue: defined as the coloration of the tongue;

**CDS** Coloration of the dorsolateral stripes: defined as the background coloration of the dorsolateral stripes;

**VLBC** Ventrolateral body coloration;

**VBC** Ventral body coloration.

The principal component analysis (PCA) was conducted using mensural data, including SVL, TAL, HL, HW, HD, SEL, FLL, HLL, T4L, and TRL, while morphological measurements for *D. flaviceps* and *D. danbaense* were sourced from Liu et al. (2023). Following size correction of the morphological measurements, PCA was performed to assess whether the newly collected specimens and species occupy distinct positions in morphospace, and to examine the congruence of these patterns with species boundaries inferred from molecular phylogenetic analyses. The PCA was performed using the prcomp command in R v. 4.3.2 (R Core Team 2025) and scatterplots were generated with the R package ggplot2 v. 3.4.4 (Wickham 2016). Considering that sexual dimorphism is acknowledged in morphometric measurements for species within the genus *Diploderma* (Wang et al. 2019a; Cai et al. 2022; Liu et al. 2023), mensural data for each sex were analyzed separately.

### ﻿Molecular data and phylogenetic analysis

Total genomic DNA was extracted from the liver tissue of every specimen collected in this study using the QIAamp DNA Mini Kit (QIAGEN, Hilden, Germany) following the manufacturer’s recommended protocols. The mitochondrial gene NADH dehydrogenase subunit 2 (*ND2*) was amplified and sequenced. The primer sequences (Jap_70F: CCACCAAACAACTACACCTA, Jap_1559R: GGATTAATGCCCTCTGGATT) were retrieved from Wang et al. (2019a). The PCR and sequencing methods followed Liu et al. (2023).

The acquired nucleotide sequences were initially subjected to forward and reverse strand proofreading and editing using SeqMan 7.1.0.44 the DNAstar 7.1 software package (DNAStar Inc., Madison, WI, USA) (Skwor 2012). There were 13 novel sequences of *ND2* obtained in this work, and a total of 103 *ND2* sequences of 44 *Diploderma* species were downloaded from GenBank for phylogenetic analyses. In addition, corresponding sequences of *Pseudocalotes brevipes* (AF128502), *Bronchocela cristatella* (KR053114), and *Laodracon carsticola* (OR544068) were downloaded from GenBank and used as outgroups (Table 1).

Sequences were aligned in MEGA X (Kumar et al. 2018) using ClustalX (Thompson et al. 1997) with default settings. Phylogenetic analyses were conducted using Bayesian inference (BI) and maximum likelihood (ML) methods implemented in MrBayes v. 3.2.7a (Ronquist et al. 2012) and MEGA X (Kumar et al. 2018), respectively. Prior to the phylogenetic analyses, the best evolutionary model was selected based on the Bayesian Information Criterion (BIC) using ModelFinder (Kalyaanamoorthy et al. 2017). The GTR + I + G4 model was selected as the best model for the mitochondrial gene. In the BI analyses, two runs were performed simultaneously with four Markov chains. The chains were run for 10,000,000 generations and sampled every 1000 generations. The first 25% of the sampled trees were discarded as burn-in, and then the remaining trees were used to estimate Bayesian posterior probabilities (BPPs); nodes with BPP values of 0.95 and higher were considered well supported (Huelsenbeck et al. 2001; Wilcox et al. 2002). In the ML analyses, sequence substitution model was selected using the auto parameter with provision for Free-Rate heterogeneity. 1000 bootstrap pseudoreplicates via the ultrafast bootstrap (UFB) approximation algorithm were used to construct a final consensus tree; nodes with UFB values of 95% and above were considered significantly supported (Minh et al. 2013). Finally, uncorrected genetic pairwise distances (*p*-distances) for *ND2* were calculated using default parameters in MEGA X (Kumar et al. 2018).

## ﻿Results

### ﻿Phylogeny

**Figure 2.**
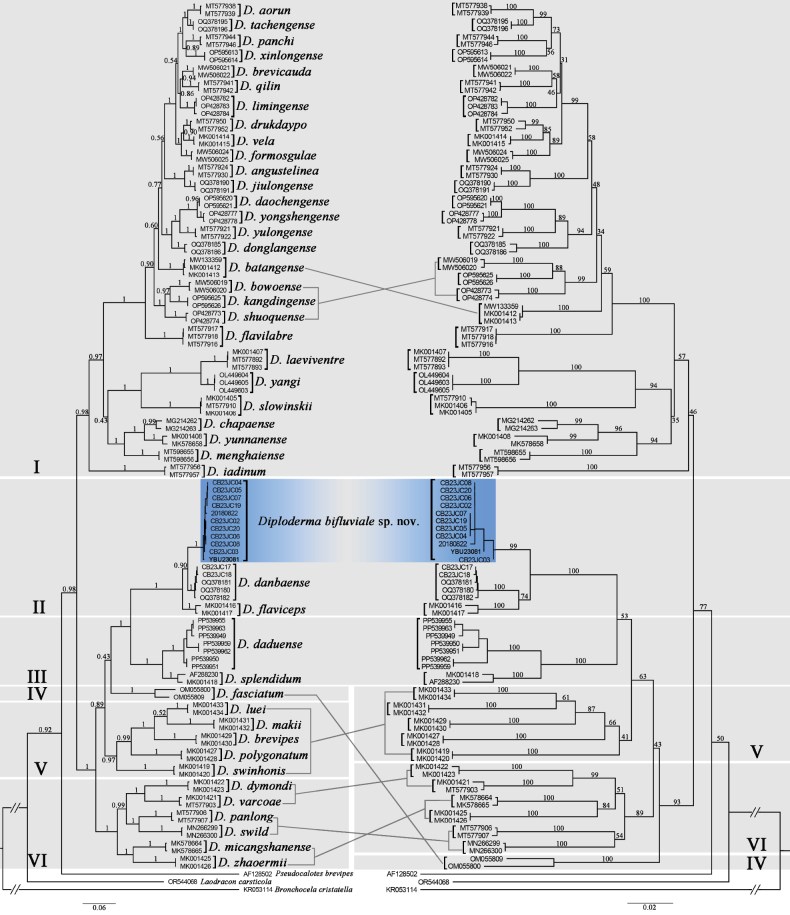
Maximum-likelihood and Bayesian inference phylogenies based on mitochondrial *ND2* genes. ML bootstrap support and Bayesian posterior probability support is denoted above each node. The left panel of this figure depicts the Bayesian (BI) tree, while the right panel presents the maximum likelihood (ML) tree.

The *ND2* sequence alignments were 984 bp in length, the BI and ML analyses produced two similar phylogenetic tree topologies, with all analyzed *Diploderma* species forming a strongly supported monophyletic group. We further identified the presence of six unique subclades within this group. The newly collected the upper Dadu River Valley population forms a distinct lineage within subclade II and constitutes a sister group with *Diploderma danbaense*, which is strongly supported by the analysis. The topological structures of the two phylogenetic trees are delineated with lines (Fig. 2), and the topological structure obtained from the Bayesian inference analysis is consistent with previous studies.

Uncorrected mean genetic distances ranged from approximately 1.36% to 27.50% across all samples, whereas the genetic distance between the population from the upper Dadu River Valley and other congeners is at least 3.88% (*D. danbaense*), which are all greater than the genetic distance (2.6%) between the two recognized species *Diploderma drukdaypo* and *Diploderma vela* (Suppl. material 1).

### ﻿Morphology

**Figure 3.**
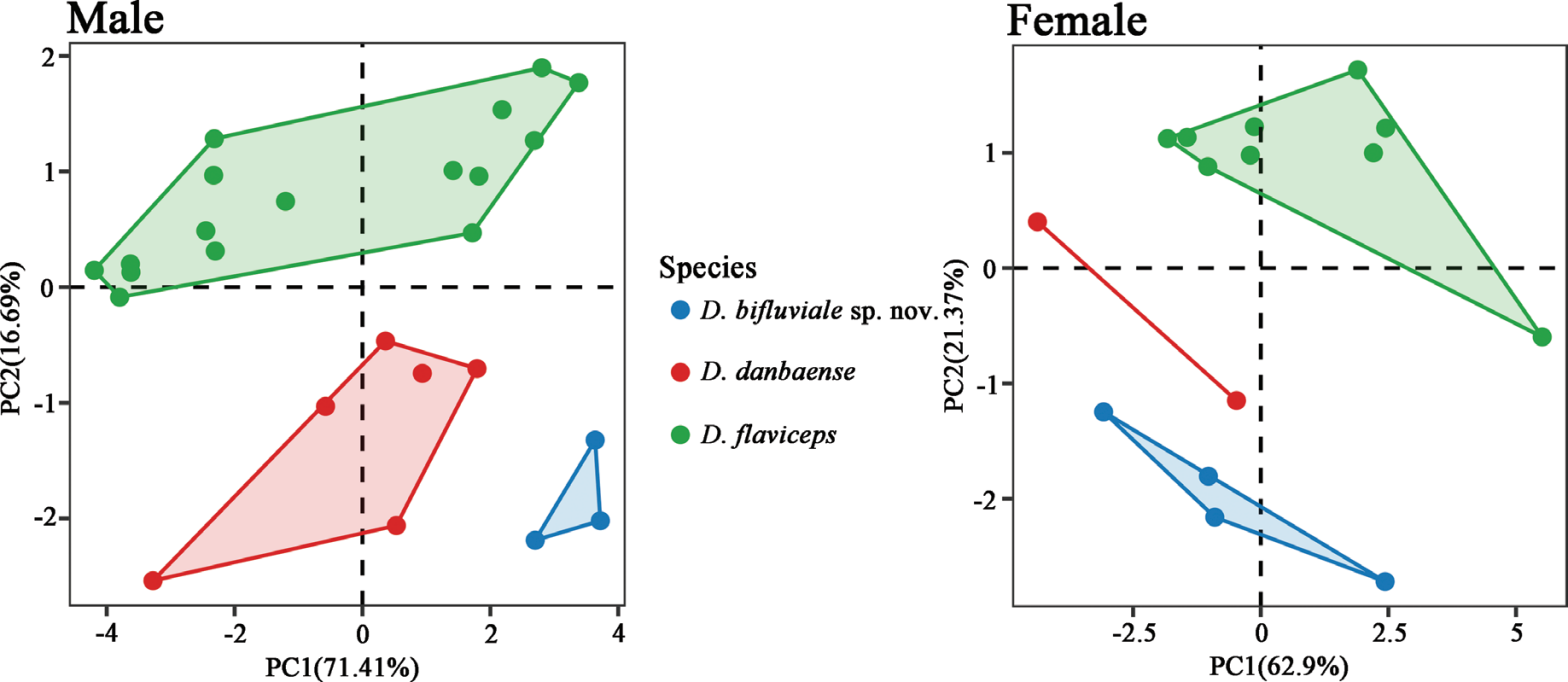
PCA based on ten morphometric characteristics (SVL, TAL, HL, HW, HD, SEL, FLL, HLL, T4L, and TRL) for *Diploderma bifluviale* sp. nov. (blue), *D. danbaense* (red), and *D. flaviceps* (green). Numbers inside the brackets indicate the percentages of the total variance explained by each axis.

Morphometric variation within subclade II species, including the upper Dadu River Valvey population, *Diploderma flaviceps*, and *D. danbaense*, was analyzed using PCA. Prior to analysis, we excluded two male *D. flaviceps* due to missing TAL data (Liu et al. 2023). and one female individual from the upper Dadu River valley population with pre-existing caudal damage upon collection. Prior to analysis, we excluded two male *D. flaviceps* and one female *Diploderma bifluviale* sp. nov. due to pre-existing caudal damage at the time of collection. For males, the first two principal components (PC1 and PC2) accounted for 71.41% and 16.69% of the variance, respectively, cumulatively explaining 88.09% of the total variance (Suppl. material 2). While PC1 showed marginal non-significance among species (*p* = 0.055), with only *D. flaviceps*-*D. bifluviale* approaching significance (*p* = 0.044), PC2 revealed extreme interspecific differentiation (*p* < 0.0001). This was driven by highly significant *D. flaviceps* contrasts with both *D. bifluviale* (*p* < 0.0001) and *D. danbaense* (*p* < 0.0001), while *D. danbaense*-*D. bifluviale* showed no differentiation (*p* = 0.437). Variables of FLL, SVL, SEL, and TAL presented high loadings on these components. For females, PC1 explained 62.9% of the variance, while PC2 added 21.37%, resulting in a cumulative variance of 84.28% (Suppl. material 3). ANOVA revealed no significant differences among species in PC1 scores (*p* = 0.222), supported by Tukey’s HSD tests (all pairwise *p* > 0.23). In contrast, PC2 scores differed significantly overall (*p* < 0.0001), with post-hoc tests indicating pronounced divergence between *D. flaviceps* and *D. bifluviale* (*p* < 0.0001), marginal significance between *D. danbaense* and *D. bifluviale* (*p* = 0.045), and a non-significant trend between *D. flaviceps* and *D. danbaense* (*p* = 0.065). The first two principal components showed higher loadings for variables of SVL, HL, TAL, and SEL. The PCA scatter plot based on morphological data reveals distinct intraspecific clustering and interspecific separation among individuals of the upper Dadu River Valley population and its two phylogenetically close congeners *D. danbaense* and *D. flaviceps*, with this pattern being consistently maintained in both male and female specimens (Fig. 3).

### ﻿Taxonomy

#### ﻿ Diploderma bifluviale sp. nov.

Taxon classificationAnimaliaSquamataAgamidae

4180A25A-0C28-5B6A-B48E-C0820DF3D4A9

https://zoobank.org/63E48A43-C4CC-4ABC-BDE7-B1CA02CCEBEC

Figs 4, 5

Japalura flaviceps authority, date: Zhao 2003: 84, partim in the Aba Tibetan and Qiang Autonomous Prefecture

##### Type material.

**Figure 4.**
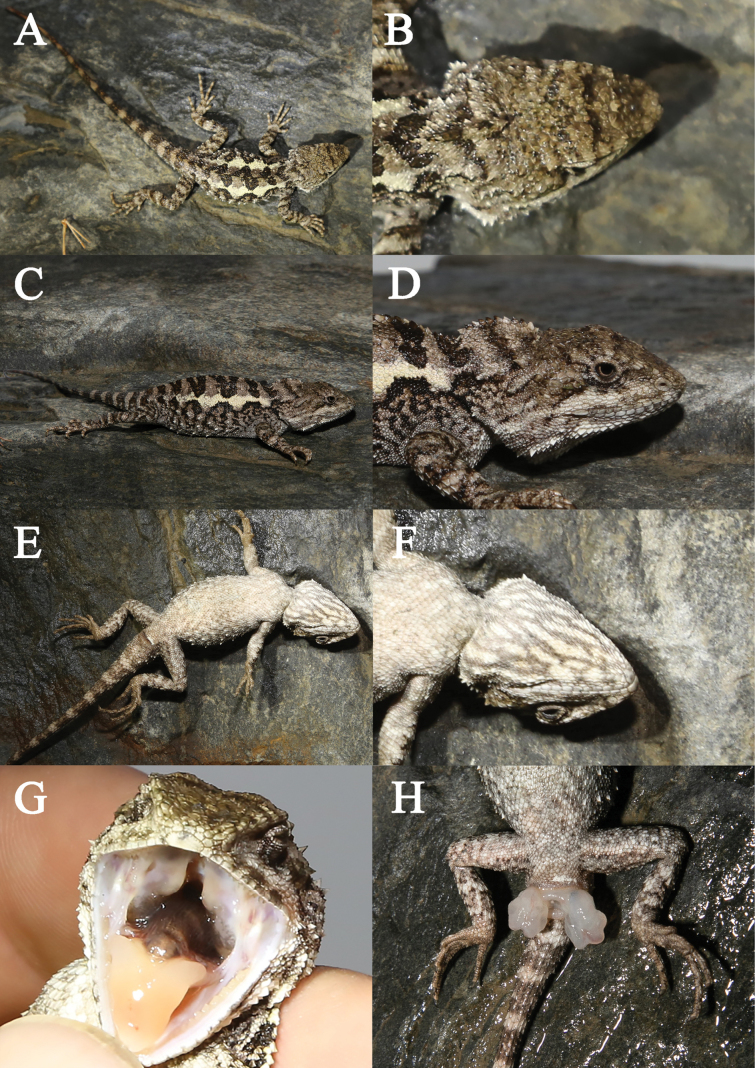
Holotype (CB23JC04) of *Diploderma bifluviale* sp. nov. in life. A. Dorsal view; B. Close up-view of the dorsal side of the head; C. Lateral view; D. Close up-view of the lateral side of the head; E. Ventral view; F. Close up-view of the ventral side of the head; G. Close-up view of the oral cavity; H. Close-up views of the femoral and precloacal regions.

**Figure 5.**
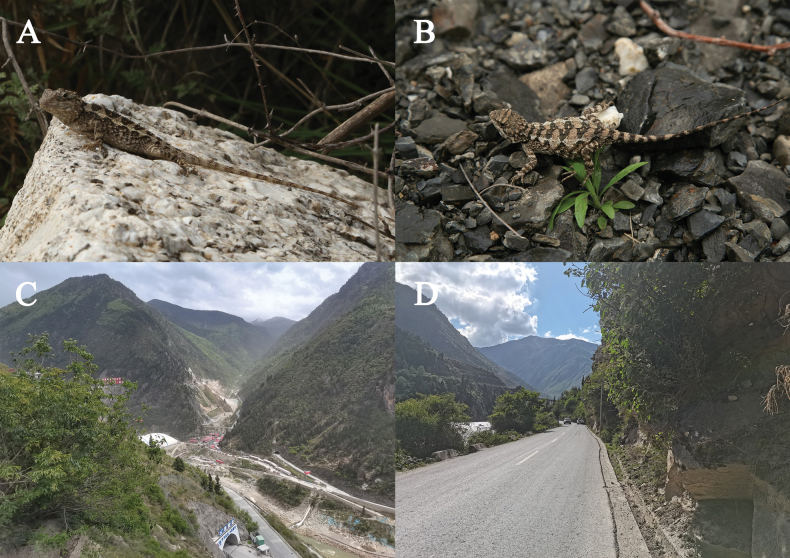
*Diploderma bifluviale* sp. nov. from near the type locality and habitats of the new species. A. Holotype CB23JC04 (male) lateral view; B. Allotype CB23JC08 (female) dorsal view; C. Distant view of the type locality of *Diploderma bifluviale* sp. nov.; D. Close view of the type locality of *Diploderma bifluviale* sp. nov.

***Holotype***: • CIB119368 (field number CB23JC04), adult male, collected from Baiwan Town (31.809249°N, 101.88182°E, 2306 m a.s.l.), Barkam City, Aba Tibetan and Qiang Autonomous Prefecture, Sichuan Province, China (Fig. 4). ***Allotype***: • CIB119369 (field number CB23JC08), adult female, also collected from Baiwan Town (31.803494°N, 101.91378°E, 2525 m a.s.l.) (Fig. 5B). ***Paratypes***: • subadult CIB-CB23JC02 and female CIB-CB23JC03 are collected from Baiwan Town (31.807989°N, 101.886555°E, 2314 m); • juvenile CIB-CB23JC05, male CIB-CB23JC06 and female CIB-CB23JC07 are collected from the same location as holotype; male CIB-CB23JC19 and female CIB-CB23JC20 are collected from Baiwan Town (31.809273°N, 101.881811°E, 2304 m a.s.l.), and juvenile CIB-201808022 was collected from Baiwan Town (31.808662°N 101.883118°E, 2290 m a.s.l.), • female YBU23081 (GP10410) was collected from Lewu Town (31.466004°N, 102.091464°E, 2252 m a.s.l.), Jinchuan County Aba Tibetan and Qiang Autonomous Prefecture, Sichuan Province, China, and subadult YBU22294 was collected from Xinsha village (31.59322°N, 102.06453°E, 2187 m a.s.l.), Jichuan County.

##### Diagnosis.

*Diploderma bifluviale* sp. nov. can be diagnosed from other *Diploderma* species by a combination of the following morphological characteristics: (1) body size short, SVL 62.51~64.47 mm, mean 63.62 mm (sample standard deviation 1.01) in adult males, 63.13~72.55 mm, mean 68.41 mm (3.53) in adult females; (2) tail short, TAL/SVL 1.41~1.53, mean 1.48 (0.06) in adult males, 1.37~1.56, mean 1.46 (0.08) in adult female; (3) head relatively long, HW/HL 0.71~0.78, mean 0.75 (0.03) in adult males, 0.68~0.74, mean 0.72 (0.02) in adult females; (4) limbs moderately long, FLL/SVL 0.42~0.44, mean 0.43 (0.01) in adult males, 0.41~0.42, mean 0.41 (0.01) in adult females, HLL/SVL 0.64~0.69, mean 0.66 (0.03) in adult males, 0.64~0.69, mean 0.65 (0.02) in adult females; (5) MD 49~54; (6) F4S 15~17, T4S 20~23; (7) tympanum concealed; (8) nuchal and dorsal crests almost continuous, scales of nuchal and dorsal crests enlarged, moderately erected skin fold under nuchal in males in life, weakly erected skin fold under nuchal crest and no skin fold under dorsal crest in females in life; (9) distinct transverse gular fold present; (10) ventral scales of head almost uniform in size, posterior and side ones smaller, all strongly keeled; (11) ventral scales of body strongly keeled; (12) gular spot absent in both sexes; (13) dorsolateral stripes distinct in males, strongly jagged and the upper and lower edges are almost touching each other, pale yellow in life; (14) a series of dark spots or a dark stripe like large wavy between dorsolateral stripes on dorsum; (15) a distinct wide black stripe on shoulder fold region on each side; (16) stripes around eye absent or very indistinct; (17) tongue wheat color in life; (18) fourth toe with claw reaching either the tympanum or the area between shoulder and tympanum when hind limbs adpressed forward (Table 2).

**Table 2.** Morphological comparison between *Diploderma bifluviale* sp. nov., *D. danbaense*, and *D. flaviceps* (range value and average value). Morphometric measurements are in the unit of mm.

	*Diploderma bifluviale* sp. nov.		*D. danbaense*		*D. flaviceps*	
	♂ (n = 3)	♀ (n = 5)	♂ (n = 6)	♀ (n = 2)	♂ (n = 18)	♀ (n = 9)
SVL	62.51~64.47 (63.62)	63.13~72.55 (68.41)	64.72~77 (70.05)	69.14~76.6 (72.87)	60.5~75.6 (68.42)	53.4~67.1 (61.78)
TAL	88.24~97.68 (93.91)	93.41~108.9 (100.43)	112.7~130 (121.10)	109.8~119.1 (114.45)	117.7~151 (135.20)	103.7~127 (116.76)
HL	19.63~20.51 (20.01)	19.48~21.48 (20.28)	21.38~26.6 (22.88)	19.52~23.5 (21.51)	18.9~24.9 (22.08)	16.3~127 (18.82)
HW	14.64~15.58 (14.96)	13.31~15.73 (14.57)	14.4~17.9 (15.81)	13.86~14.7 (14.28)	14.6~20.6 (17.38)	12.6~15.6 (13.97)
HD	11.63~12.94 (12.30)	9.68~13.36 (11.61)	10.87~15 (13.11)	11.5~12.8 (12.15)	10.7~14.8 (12.88)	9.6~12.5 (10.90)
SEL	7.85~9.28 (8.56)	8.39~9.02 (8.71)	7.5~9.9 (8.68)	8.4~8.78 (8.59)	5.9~8.9 (7.42)	5.2~6.8 (6.17)
FLL	26.24~28.16 (27.05)	25.69~29.83 (28.38)	28.7~33.1 (31.42)	28.44~33.6 (31.02)	28.8~36.1 (32.37)	24.3~31.6 (28.53)
HLL	40.37~44.2 (41.99)	40.2~47.24 (44.72)	45.73~53.5 (48.83)	43.9~50 (46.95)	46.3~56.4 (51.87)	37.2~50.1 (45.81)
T4L	10.52~10.67 (10.58)	9.39~11.39 (10.78)	10.01~12.4 (11.79)	11.17~12.1 (11.64)	11.4~14.9 (13.11)	9.6~11.7 (11.13)
TRL	28.88~29.66 (29.15)	30.67~35.35 (33.96)	28.78~33.2 (31.05)	32.55~34.5 (33.53)	26.7~34.6 (30.86)	24.3~33 (28.98)
TAL/SVL	1.41~1.53 (1.48)	1.37~1.56 (1.46)	1.61~1.89 (1.73)	1.55~1.59 (1.57)	1.88~2.09 (1.99)	1.72~2.17 (1.90)
SEL/HL	0.40~0.45 (0.43)	0.42~0.44 (0.43)	0.35~0.43 (0.38)	0.36~0.45 (0.40)	0.31~0.37 (0.34)	0.3~0.34 (0.33)
HW/HL	0.71~0.78 (0.75)	0.68~0.74 (0.72)	0.64~0.75 (0.69)	0.63~0.71 (0.67)	0.76~0.84 (0.79)	0.71~0.78 (0.74)
HD/HW	0.79~0.88 (0.82)	0.73~0.86 (0.80)	0.74~0.90 (0.83)	0.83~0.87 (0.85)	0.7~0.78 (0.74)	0.75~0.83 (0.78)
FLL/SVL	0.42~0.44 (0.43)	0.41~0.42 (0.41)	0.41~0.48 (0.45)	0.41~0.44 (0.42)	0.44~0.49 (0.47)	0.43~0.49 (0.46)
HLL/SVL	0.64~0.69 (0.66)	0.64~0.69 (0.65)	0.66~0.71 (0.70)	0.63~0.65 (0.64)	0.72~0.8 (0.76)	0.7~0.81 (0.74)
TRL/SVL	0.45~0.46 (0.46)	0.48~0.52 (0.50)	0.43~0.46 (0.44)	0.45~0.47 (0.46)	0.41~0.49 (0.45)	0.44~0.5 (0.47)
MD	50~54 (52.33)	49~50 (49.60)	47~53 (49.33)	49~58 (56.00)	40~55 (46.12)	46~54 (50.00)
VN	63~64 (63.33)	62~64 (62.80)	61~72 (65.83)	64~74 (69.00)	−	−
SL	9~11	8~11	9~10	9~11	9~11	9~11
IL	10~13	9~12	10~11	9~12	10–12	10~12
NSL	1~2	1~2	1~2	2	1~2	1~2
F4S	16~16	15~17	16~20	17~20	16~20	16~18
T4S	21~21	20~23	21~26	21~26	22~26	22~27
SOR	3~4	3~4	3~5	4~5	3~5	3~5

##### Description of holotype.

Adult male, body relatively small-sized, SVL 62.5 mm; tail short, TAL 88.2mm; head longer (19.9 mm) than wide (15.6 mm); head depth 12.3 mm; length of tallest nuchal crest 0.5 mm; snout–eye length 8.6 mm; foreleg length 26.2 mm; hindleg length 40.4 mm; toe IV length 10.5 mm; trunk length 28.5 mm; snout moderately long, SEL/HL 0.43. Rostral flat, bordered by six small postrostral scales; dorsal head scales heterogeneous in size, most of them keeled. Number of scales between supraoculars 16; nasal scale approximately oval; internasals 9; loreals 7/6, small, unkeeled; mental pentagonal; supralabials 10/10, infralabial scales 11/13, supratemporals 4/3, and middorsal crest scales 54, keeled; gular scales 30 and ventralscales 64; finger IV subdigital lamellae 16/17; toe IV subdigital lamellae 21/22; nasal supralabial scale rows 1/1; suborbital scale rows 3/4. Transverse gular fold present; gular pouch present in life; tympanum concealed, covered with small keeled scales; well-developed skin fold under nuchal crest present, vertebral crest continuous between nuchal and dorsal sections; axillary scales much smaller than remaining dorsals; dorsal and ventral scales distinctively keeled exclude scales around eyes and lips; dorsal scales of head, trunk, limbs, and tail heterogeneous in size, ventral scales of head almost ventral scales of head almost uniform in size with few larger scales, posterior and side ones smaller, all strongly keeled; fold present in front of shoulder; fourth toe with claw reaching at tympanum when hindlimbs adpressed forward. Upper and lower edges of dorsolateral stripes are strongly serrated and separated only by a lemon-chiffon dorsal scale at the serrated tip; tail scales all strongly keeled, ventral tail scales slightly larger than dorsal tail scales; fourth toe with claw reaching tympanum when hind limbs adpressed forward (Table 2).

##### Coloration in life.

The dorsal head is mainly dark khaki (189, 183, 107), with an olive (128, 128, 0) transverse stripe at the front and a blurred olive stripe at the back connecting the left and right supraoculars, and olive markings on the anterodorsal edge. Olive dots walking on the rest of dorsal head. Lateral surfaces of head grey (128, 128, 128) to dark khaki. There are seven stripes around the upper eye except the subocular regions on each side, with a thick black and dark khaki stripe extending from the posterior nasal through the lower palpebral to the temporal region. The subocular scale rows are white-smoke (245, 245, 245) with few dark grey (169, 169, 169) scales. The supralabials and infralabials are white smoke with dark grey, make the eye area appear inconspicuous radial lines. The inner-lip coloration is smoky white, and the coloration of the tongue wheat (245, 222, 179).

The dorsal surface is predominantly dark khaki. A lemon-chiffon (255, 250, 205) strongly jagged dorsolateral stripe from neck to pelvis on each side of body. At the serrated tip, the upper and lower edges of these stripes are separated only by a lemon-chiffon dorsal scale. Between the dorsolateral stripes there is an almost continuous black to olive almost wavy pattern. A distinct wide black stripe on shoulder fold region on each side. Some reticular markings below dorsolateral stripe on each side of body, with dark-khaki patches wrapped around a larger white (255, 255, 255) dorsal scale on each side of body. Dorsal surfaces of limbs dark khaki with black to olive trans verse bands. Dorsal surface of tail wheat with olive transverse bands.

Ventral surface of head white-smoke with dim grey (105, 105, 105) reticulated pattern. No gular spot. Ventral surfaces of body and limbs white-smoke with dim grey with smoke pattern, ventral surface of tail white-smoke with indistinct dark khaki transverse bands (Fig. 4).

##### Variations.

*Diploderma bifluviale* sp. nov. is sexually dimorphic: (1) males are smaller than females, SVL 62.51~64.47 (mean 63.62) mm vs 63.13~72.55 (mean 68.41) mm; (2) trunk relatively shorter, TRL/SVL 0.45~0.46 (mean 0.46) vs 0.48~0.52 (mean 0.50); (3) fore-limb relatively longer, FLL/SVL 0.42~0.44 (mean 0.43) vs 0.41~0.42 (mean 0.41); (4) skin folds under nuchal and dorsal crest obviously present in adult males only; (5) dorsolateral stripes prominent present in adult males, absent or not noticeable in females.

##### Comparisons.

*Diploderma bifluviale* sp. nov. differs from *D. flaviceps* by having the following combined characteristics: (1) relatively shorter body (SVL 62.51~64.47 [mean 63.62] mm vs 60.5~75.6 [mean 68.42] mm in males; (2) relatively shorter tail (TAL/SVL 1.41~1.53 vs 1.88~2.09 in males, 1.37~1.56 vs 1.72~2.17 in females); (3) relatively shorter fore-limbs (FLL/SVL 0.42~0.44 [mean 0.43] vs 0.44~0.49 [mean 0.47] in males; (4) relatively shorter hind limbs (HLL/SVL 0.64~0.69 [mean 0.66] vs 0.72–0.80 [mean 0.76] in males, 0.64~0.69 [mean 0.65] vs 0.7~0.81 [mean 0.74] in females; (5) relatively longer snout–eye length (SEL/HL 0.42~0.44 [mean 0.43] vs 0.30~0.34 [mean 0.33] in females (Table 2); (6) moderately erected skin fold under dorsal crest in males in life (vs strongly erected) and the absence of a skin fold under dorsal crest in females in life (vs presence); (7) upper and lower edges of dorsolateral stripes are separated only by a lemon-chiffon dorsal scale at the serrated tip in males (vs relatively smooth or wavy and separated by more than 3 dorsal scales), dorsolateral stripes absent or not noticeable in females (vs distinct); (8) tongue wheat color in life (vs ‘pale flesh’ [240, 208, 202]); (9) fourth toe with claw reaching either the tympanum or the area between shoulder and tympanum when hind limbs adpressed forward (vs reaching the posterior edge of the eye).

*Diploderma bifluviale* sp. nov. differs from *D. danbaense* by having the following combined characteristics: (1) relatively shorter body (SVL 62.51~64.47 [mean 63.62] mm vs 64.72~77 [mean 70.05] mm in males; (2) relatively shorter tail (TAL/SVL 1.41~1.53 vs 1.61~1.89 in males, 1.37~1.56 vs 1.55~1.59 in females); (3) relatively shorter head (HL 19.63~20.51 [mean 20.01] mm vs 21.38~26.6 [mean 22.88]) in males; (4) relatively shorter hind limbs (HLL/SVL 0.64~0.69 [mean 0.66] vs 0.66~0.71 [mean 0.70] in males; (5) relatively longer trunk length (TRL/SVL 0.48~0.52 [mean 0.50] vs 0.45~0.47 [mean 0.46]) in adult females (Table 2); (6) upper and lower edges of dorsolateral stripes are separated only by a lemon-chiffon dorsal scale at the serrated tip in males (vs relatively smooth or wavy and separated by 3 [fewer 1] dorsal scales); (7) tongue wheat color in life (vs ‘pale flesh’); (8) fourth toe with claw reaching either the tympanum or the area between shoulder and tympanum when hind limbs adpressed forward (vs the area between eyes and ears).

*Diploderma bifluviale* sp. nov. differs from *D. daduense* (Cai, Liu & Chang, 2024) by having the following combined characteristics: (1) relatively shorter body (SVL 62.51~64.47 [mean 63.62] mm vs 74.7–95.0 [mean 86.5] mm in males; (2) relatively shorter tail (TAL/SVL 1.41~1.53 vs 2.04~2.62 in males; (3) dorsolateral stripes are lemon-chiffon (vs green-yellow anteriorly, cyan in the center, and blurry off-white posteriorly); (4) tongue wheat color in life (vs ‘pale flesh’).

*Diploderma bifluviale* sp. nov. differs from *D. brevipes* (Gressitt, 1936), *D. chapaense* (Bourret, 1937), *D. fasciatum* (Mertens, 1926), *D. hamptoni* (Smith, 1935), *D. luei* (Ota, Chen & Shang, 1998), *D. makii* (Ota, 1989), *D. menghaiense* Liu, Hou, Wang, Ananjeva & Rao, 2020, *D. micangshanense* (Song, 1987), *D. nangunhe* Liu, Li, (Yang, Hou, Rao & Ananjeva, 2024), *D. ngoclinense* (Ananjeva, Orlov & Nguyen, 2017), *D. polygonatum* Hallowell, 1861, *D. swinhonis* (Günther, 1864), and *D. yunnanense* (Anderson, 1878) by the presence of a transverse gular fold (vs absence).

*Diploderma bifluviale* sp. nov. differs from *D. dymondi* (Boulenger, 1906), *D. panlong* Wang, Che & Siler, 2020, *D. slowinskii* (Rao, Vindum, Ma, Fu & Wilkinson, 2017), *D. swild* Wang, Wu, Jiang, Chen, Miao, Siler & Che, 2019, and *D. varcoae* (Boulenger, 1918) by having concealed tympana (vs exposed).

*Diploderma bifluviale* sp. nov. differs from *D. angustelinea* Wang, Ren, Wu, Che & Siler, 2020, *D. aorun* Wang, Jiang, Zheng, Xie, Che & Siler, 2020, *D. batangense* (Li, Deng, Wu & Wang, 2001), *D. bowoense* Wang, Gao, Wu, Siler & Che, 2021, *D. brevicauda* (Manthey, Denzer, Hou & Wang, 2012), *D. chapaense* (Bourret, 1937), *D. daochengense* Cai, Zhang, Li, Du, Xie, Hou, Zhou & Jiang, 2022, *D. donglangense* Liu, Hou, Ananjeva & Rao, 2023, *D. flavilabre* Wang, Che & Siler, 2020, *D. formosgulae* Wang, Gao, Wu, Dong, Shi, Qi, Siler & Che, 2021, *D. iadinum* (Wang, Jiang, Siler & Che, 2016), *D. jiulongense* Liu, Hou, Ananjeva & Rao, 2023, *D. kangdingense* Cai, Zhang, Li, Du, Xie, Hou, Zhou & Jiang, 2022, *D. laeviventre* (Wang, Jiang, Siler & Che, 2016), *D. limingense* Liu, Hou, Rao & Ananjeva, 2022, *D. nangunhe* Liu, Li, Yang, Hou, Rao & Ananjeva, 2024, *D. panchi* Wang, Zheng, Xie, Che & Siler, 2020, *D. qilin* Wang, Ren, Che & Siler, 2020, *D. splendidum* (Barbour & Dunn, 1919), *D. tachengense* Liu, Hou, Ananjeva & Rao, 2023, *D. xinlongense* Cai, Zhang, Li, Du, Xie, Hou, Zhou & Jiang, 2022, *D. yangi* Wang, Zhang & Li, 2022, *D. yongshengense*, *D. yulongense*, and *D. zhaoermii* (Gao & Hou, 2002) by the absence of a gular spot in males in life (vs presence of a colourful gular spot).

*Diploderma bifluviale* sp. nov. differs from *D. drukdaypo* (Wang, Ren, Jiang, Zou, Wu, Che & Siler, 2019) by having strongly keeled ventral scales of body (vs smooth or weakly keeled); from *D. qiaojiaense* (Liu, Hou & Rao, 2024) by having lemon-chiffon dorsolateral stripes in males in life (vs light green); from *D. grahami* (Stejneger, 1924) due to having relatively longer hind limbs (HLL/SVL 0.64~0.69 vs 0.61), and the presence of dorsolateral stripes (vs absence) in males; from *D. kangdingense* Cai, Zhang, Li, Du, Xie, Hou, Zhou & Jiang, 2022 by having dim grey ventrolateral surface of body in males in life (vs yellow); from *D. shuoquense* Liu, Hou, Rao & Ananjeva, 2022 by having strongly keeled ventral head scales and from *D. vela* (Wang, Jiang & Che, 2015) by having inconspicuous radial lines around the eyes (vs distinct radial stripes), and having moderately erected skin fold under nuchal (vs a pronounced, sail-like, and continuous vertebral crest) in males.

##### Distribution and natural history.

*Diploderma bifluviale* sp. nov. is currently known to inhabit the semi-arid region of warm-dry valley in the upper reaches of the Dadu River. It is primarily concentrated around Shuangjiangkou, located at the confluence of the Chuosijia River and the Jiaomuzu River, spanning Jinchuan County and Barkam City (Ma’erkang City) within the Aba Tibetan and Qiang Autonomous Prefecture, Sichuan Province, China (Fig. 1). This area is characterized by long hours of sunlight, frequent clear days, distinct wet and dry seasons, and significant diurnal temperature variations (Zhang 1992). The known distribution range of *Diploderma bifluviale* sp. nov. exhibits higher humidity and greater vegetation coverage compared to that of *D. danbaense*.

The species is known to inhabit altitudes ranging from 2187 to 2314 meters, residing in arid shrublands with small leaves and scattered rock piles, where the shrubs can reach heights of 0.5–2 meters (Fig. 5). All specimens were collected between 09:00 and 18:00 hr from June to August. During the investigation, it was observed that this species preys on valley insects, this species represents one of the few secondary consumers inhabiting this arid river valley ecosystem.

This species is oviparous. Specimens CB23JC16, CIB119369, CB23JC07, CB23JC03, and CB23JC20 were found to contain 3, 5, 6, 6, and 7 eggs, respectively.

##### Etymology.

The specific epithet *bifluviale* is derived from the Latin words *bi*- meaning two, and *fluviale*, relating to rivers. This name refers to the species’ discovery location near Shuangjiangkou (双江口), which denotes the area around the confluence of the Chuosijia River (绰斯甲河, Chuosi River) and the Jiaomuzu River (脚木足河, Kyom-kyo River) of Sichuan Province, China. We suggest Upper Dadu Mountain Lizard as its English common name and 双江口攀蜥 (Chinese phonetic alphabet: Shuāng jiāng kǒu Pānxī) as its Chinese common name.

## ﻿Discussion

Based on integrated phylogenetic analysis and the PCA of morphological data, we propose that the *Diploderma* species from the upper Dadu River Valley represents a distinct species. The species delimitation employing *ND2* gene-based phylogenetic reconstruction has been extensively validated (Wang et al. 2019b; Cai et al. 2022, 2024; Liu et al. 2023, 2024a, b), although minor topological discrepancies were observed between phylogenetic trees generated by two different algorithmic approaches (BI and ML). These variations likely reflect inherent methodological differences between BI and ML analytical frameworks (Yang 1994; Yang and Rannala 1997). Notably, in subclade II, both phylogenetic analyses consistently and unambiguously support the status of *Diploderma bifluviale* sp. nov. as a distinct species. Future studies with more genes and species will help strengthen the evolutionary relationships within the *Diploderma* genus. PCA further demonstrates that *Diploderma bifluviale* sp. nov. is morphometrically distinct from its two phylogenetically closest congeners, *D. danbaense* and *D. flaviceps*.

*Diploderma bifluviale* sp. nov. is the northernmost species within the *D. flaviceps* group, inhabiting shrublands on both sides of the upper Dadu River’s warm-dry valleys. Through several surveys conducted by us from 2018 to 2024, only a few individuals were observed on three occasions, indicating that this species is rare and may constitute a very small population. The discovery of this novel species carries substantial conservation significance for informing future biodiversity preservation strategies.

The known concentrated distribution area of this species has been partially submerged by the Shuangjiangkou Reservoir. This reservoir serves as a controlling reservoir for the upper reaches of the Dadu River mainstream and possesses annual regulation capabilities. The dam area is located approximately 2–6 kilometers downstream from the confluence of the Chuosijia River and the Jiaomuzu River in Barkam City and Jinchuan County, Aba Prefecture. Currently, the main structure of the reservoir has been completed, and initial water storage began in November 2024.

According to the relevant laws and regulations concerning ecological protection in China, the construction of hydropower stations follows a series of stringent procedures. However, practical implementation may sometimes fall short due to shorter project execution cycles, limited funding, and significant time pressures on construction schedules, which can lead to insufficient time allocated for biodiversity baseline surveys, EIA investigations, and ecological remediation. Consequently, some species within the region may be overlooked, and ecological compensation measures are frequently simplified. The timing of water storage often conflicts with biological rhythms, not avoiding critical periods for bird breeding or reptile hibernation.

To address these issues, it is recommended that relevant departments allocate sufficient time for biodiversity baseline surveys, environmental impact assessment investigations, and ecological remediation. Full consideration should be given to the impacts of reservoir construction on local native species, particularly endemic ones. Water storage schedules should respect biological rhythms, avoiding periods crucial for spawning and hibernation. Such an approach would mitigate adverse effects on wildlife, allowing them to adapt swiftly and migrate autonomously to areas above the new waterline, thereby adhering more closely to ecological management laws and principles of ecological protection.

### ﻿Comparative specimens examined

***Diploderma flaviceps***: Sanhe Township, Luding County (YBU23217), Kongyu Township, Luding County (YBU22717), Marr Village, Danba County (YBU23222), Pengba, Luding County (KIZ 057784, KIZ 84001, KIZ 84003, KIZ 057785), Shelian, Kangding City (KIZ 820065, KIZ 820052, KIZ 820068, KIZ 820069, KIZ 820054, KIZ 820049, KIZ 820070, KIZ 820047, KIZ 820050, KIZ 820055, KIZ 820043, KIZ 820046, KIZ 820081, KIZ 820056, KIZ 820045, KIZ 820059, KIZ 820048, KIZ 820057, KIZ 820067, KIZ 820061, KIZ 820071, KIZ 820044, KIZ 820058).

***Diploderma danbaense***: Bawang Township, Danba County (CIB-CB23JC17, CIB-CB23JC18, KIZ2022048, KIZ2022049, KIZ2022050, KIZ2022056, KIZ2022051), Geshiza Township, Danba County (YBU231989).

## Supplementary Material

XML Treatment for Diploderma bifluviale

## ﻿Additional information

### Use of AI

No use of AI was reported.

### Author ORCIDs

Fengjing Liu https://orcid.org/0009-0002-7280-1416

Yayong Wu https://orcid.org/0000-0003-2752-4085

Guang Yang https://orcid.org/0000-0001-6285-6937

Shuo Liu https://orcid.org/0000-0001-7825-3006

Xue Chen https://orcid.org/0000-0002-1953-1333

Jiang Chang https://orcid.org/0000-0001-7064-9321

Bo Cai https://orcid.org/0000-0002-4214-9060

### Data availability

All of the data that support the findings of this study are available in the main text or Supplementary Information.

## Supplementary materials

Supplementary material 1 Uncorrected genetic pairwise distances (*p*-distances) (%) between *Diploderma* species in this study based on the mitochondrial ND2 gene sequences Data type xlsx https://binary.pensoft.net/file/1408459This dataset is made available under the Open Database License (http://opendatacommons.org/licenses/odbl/1.0/). The Open Database License (ODbL) is a license agreement intended to allow users to freely share, modify, and use this Dataset while maintaining this same freedom for others, provided that the original source and author(s) are credited.Fengjing Liu, Yayong Wu, Jindong Zhang, Guang Yang, Shuo Liu, Xue Chen, Jiang Chang, Qiang Xie, Bo Cai

Supplementary material 2 Summary statistics of PCA analyses of males' morphometric characteristics in selected members Data type xlsx https://binary.pensoft.net/file/1408460This dataset is made available under the Open Database License (http://opendatacommons.org/licenses/odbl/1.0/). The Open Database License (ODbL) is a license agreement intended to allow users to freely share, modify, and use this Dataset while maintaining this same freedom for others, provided that the original source and author(s) are credited.Fengjing Liu, Yayong Wu, Jindong Zhang, Guang Yang, Shuo Liu, Xue Chen, Jiang Chang, Qiang Xie, Bo Cai

Supplementary material 3 Summary statistics of PCA analyses of females' morphometric characteristics in selected members Data type xlsx https://binary.pensoft.net/file/1408461This dataset is made available under the Open Database License (http://opendatacommons.org/licenses/odbl/1.0/). The Open Database License (ODbL) is a license agreement intended to allow users to freely share, modify, and use this Dataset while maintaining this same freedom for others, provided that the original source and author(s) are credited.Fengjing Liu, Yayong Wu, Jindong Zhang, Guang Yang, Shuo Liu, Xue Chen, Jiang Chang, Qiang Xie, Bo Cai

## References

[B1] Cai B (2025) Class Reptilia in Catalogue of Life China: 2025 Annual Checklist [Online]. Species 2000 China Node. Beijing. http://www.sp2000.org.cn/browse/browse_taxa [accessed on 20 May 2025]

[B2] Cai B Zhang MH Li J Du SM Xie F Hou M Zhou HM Jiang JP (2022) Three new species of *Diploderma* Hallowell, 1861 (Reptilia: Squamata: Agamidae) from the Shaluli Mountains in Western Sichuan, China. Asian Herpetological Research 13(4): 205–223. 10.16373/j.cnki.ahr.220040

[B3] Cai B Liu F Liang D Hou M Zhou H Zhong J Li J Chang J (2024) A new species of *Diploderma* (Squamata, Agamidae) from the Valley of Dadu River in Sichuan Province, with a Redescription of Topotypes of *D. splendidum* from Hubei Province, China. Animals 14(9): e1344. 10.3390/ani14091344 PMC11083360 38731347

[B4] Che J Jiang K Yan F Zhang YP (2021) Amphibians and Reptiles in Tibet: Diversity and Evolution. Science Press, Beijing, China, 459 pp. [In Chinese]

[B5] Feng KY (2019) Analysis on the environmental problems of soil erosion in the development of Dadu River Hydropower Station. Shuili Shuidian Jishu 50(S2): 229–231. https://link.cnki.net/doi/10.13928/j.cnki.wrahe.2019.S2.042

[B6] Grismer LL Wood PLJ Lee CH Quah ESH Anuar S Ngadi E Sites JWJ (2015) An integrative taxonomic review of the agamid genus *Bronchocela* (Kuhl, 1820) from Peninsular Malaysia with descriptions of new montane and insular endemics. Zootaxa 3948(1): 1–23. 10.11646/zootaxa.3948.1.1 25947760

[B7] Huelsenbeck JP Ronquist F Nielsen R Bollback JP (2001) Bayesian inference of phylogeny and its impact on evolutionary biology. Science 294(5550): 2310–2314. 10.1126/science.1065889 11743192

[B8] Kalyaanamoorthy S Minh BQ Wong TKF von Haeseler A Jermiin LS (2017) ModelFinder: Fast model selection for accurate phylogenetic estimates. Nature Methods 14(6): 587–589. 10.1038/nmeth.4285 28481363 PMC5453245

[B9] Kumar S Stecher G Li M Knyaz C Tamura K (2018) MEGA X: Molecular Evolutionary Genetics Analysis across computing platforms. Molecular Biology and Evolution 35(6): 1547–1549. 10.1093/molbev/msy096 29722887 PMC5967553

[B10] Liu S Hou M Wang J Ananjeva NB Rao DQ (2020) A new species of *Diploderma* (Squamata: Sauria: Agamidae) from Yunnan Province, China. Russian Journal of Herpetology 27(3): 127–148. 10.30906/1026-2296-2020-27-3-127-148

[B11] Liu S Hou M Ananjeva NB Rao D (2023) Four new species of the genus *Diploderma* Hallowell, 1861 (Squamata, Agamidae) from China. ZooKeys 1148: 167–207. 10.3897/zookeys.1148.97706 37235140 PMC10207273

[B12] Liu S Hou M Rao D (2024a) A New Species of *Diploderma* Hallowell, 1861 (Reptilia, Squamata, Agamidae) from Northeastern Yunnan Province, China. Taxonomy 4(2): 412–431. 10.3390/taxonomy4020020

[B13] Liu S Li Z Yang T Hou M Rao D Ananjeva NB (2024b) A new species of *Diploderma* (Squamata: Sauria: Agamidae) from Yunnan Nangunhe National Nature Reserve, China. Russian Journal of Herpetology 31(6): 379–391. 10.30906/1026-2296-2024-31-6-379-391

[B14] Macey JR Schulte JA II Larson A Ananjeva NB Wang Y Pethiyagoda R Rastegar-Pouyani N Papenfuss TJ (2000) Evaluating trans-Tethys migration: An example using acrodont lizard phylogenetics. Systematic Biology 49(2): 233–256. 10.1093/sysbio/49.2.233 12118407

[B15] McGuire JA Heang KB (2001) Phylogenetic systematics of Southeast Asian flying lizards (Iguania: Agamidae: Draco) as inferred from mitochondrial DNA sequence data. Biological Journal of the Linnean Society. Linnean Society of London 72(2): 203–229. 10.1111/j.1095-8312.2001.tb01312.x

[B16] Minh BQ Nguyen MAT von Haeseler A (2013) Ultrafast Approximation for Phylogenetic Bootstrap. Molecular Biology and Evolution 30(5): 1188–1195. 10.1093/molbev/mst024 23418397 PMC3670741

[B17] R Core Team (2025) R: A language and environment for statistical computing. R Foundation for Statistical Computing, Vienna. http://www.R-project.org/ [accessed on 5 Jan 2025]

[B18] Ronquist F Teslenko M van der Mark P Ayres DL Darling A Höhna S Larget B Liu L Suchard MA Huelsenbeck JP (2012) MrBayes 3.2: Efficient bayesian phylogenetic inference and model choice across a large model space. Systematic Biology 61(3): 539–542. 10.1093/sysbio/sys029 22357727 PMC3329765

[B19] Sitthivong S Brakels P Xayyasith S Maury N Idiiatullina S Pawangkhanant P Wang K Nguyen TV Poyarkov NA (2023) Hiding on jagged karst pinnacles: A new microendemic genus and species of a limestone-dwelling agamid lizard (Squamata: Agamidae: Draconinae) from Khammouan Province, Laos. Zoological Research 44(6): 1039–1051. 10.24272/j.issn.2095-8137.2023.062 37872005 PMC10802101

[B20] Skwor T (2012) The use of DNASTAR lasergene educational software with molecular techniques to support bacterial identification. Proceedings of the 33^rd^ Conference of the Association for Biology Laboratory Education (ABLE) 33: 327–334. http://www.ableweb.org/volumes/vol-33/?art=38

[B21] Thompson JD Gibson TJ Plewniak F Jeanmougin F Higgins DG (1997) The CLUSTAL_X Windows Interface: Flexible Strategies for multiple sequence alignment aided by quality analysis tools. Nucleic Acids Research 25(24): 4876–4882. 10.1093/nar/25.24.4876 9396791 PMC147148

[B22] Wang K Jiang K Wang YF Poyarkov Jr NA Che J Siler CD (2018) Discovery of *Japalura chapaensis* Bourret, 1937 (Reptilia: Squamata: Agamidae) from Southeast Yunnan Province, China. Zoological Research 39(2): 105–113. 10.24272/j.issn.2095-8137.2017.064 29515092 PMC5885388

[B23] Wang K Che J Lin S Deepak V Aniruddha D-R Jiang K Jin J Chen H Siler CD (2019a) Multilocus phylogeny and revised classification for mountain dragons of the genus *Japalura**s.l.* (Reptilia: Agamidae: Draconinae) from Asia. Zoological Journal of the Linnean Society 185(1): 246–267. 10.1093/zoolinnean/zly034

[B24] Wang K Ren J Jiang K Wu J Yang C Xu H Messanger K Lei K Yu H Yang J Siler CD Li J Che J (2019b) Revised distributions of some species in the genus *Diploderma* (Reptilia: Agamidae) in China. Sichuan Journal of Zoology 38(5): 481–495. https://link.cnki.net/urlid/51.1193.Q.20190815.0932.002

[B25] Wang K Wu JW Jiang K Chen JM Miao BF Siler CD Che J (2019c) A new species of Mountain Dragon (Reptilia: Agamidae: *Diploderma*) from the *D. dymondi* complex in southern Sichuan Province, China. Zoological Research 40(5): 456–465. 10.24272/j.issn.2095-8137.2019.034 31502428 PMC6755113

[B26] Wang K Ren J Wu J Chen X Jiang K Che J Siler CD (2020) Systematic revision of mountain dragons (Reptilia: Agamidae: *Diploderma*) in China, with descriptions of six new species and discussion on their conservation. Journal of Zoological Systematics and Evolutionary Research 59(1): 222–263. 10.1111/jzs.12414

[B27] Wang K Gao W Wu J Dong W Feng X Shen W Jin J Shi X Qi Y Siler CD Che J (2021) Two new species of *Diploderma* Hallowell, 1861 (Reptilia: Squamata: Agamidae) from the Hengduan Mountain Region in China and rediscovery of *D. brevicaudum* (Manthey, Wolfgang, Hou, Wang, 2012). Zootaxa 4941(1): 1–27. 10.11646/zootaxa.4941.1.1 33756946

[B28] Wang K Gao W Wu Y Che J (2024) Current Status and Future Perspectives on the Research of the Genus *Diploderma* (Reptilia: Agamidae). Dongwuxue Zazhi 59: 129–131. 10.13859/j.cjz.202424012

[B29] Wickham H (2016) ggplot2: Elegant Graphics for Data Analysis. Springer, New York. https://ggplot2.tidyverse.org/ [accessed on 5 Jan 2025] 10.1007/978-3-319-24277-4_9

[B30] Wilcox TP Zwickl DJ Heath TA Hillis DM (2002) Phylogenetic relationships of the Dwarf Boas and a comparison of Bayesian and bootstrap measures of phylogenetic support. Molecular Phylogenetics and Evolution 25(2): 361–371. 10.1016/S1055-7903(02)00244-0 12414316

[B31] Yang Z (1994) Maximum likelihood phylogenetic estimation from DNA sequences with variable rates over sites: Approximate methods. Journal of Molecular Evolution 39(3): 306–314. 10.1007/BF00160154 7932792

[B32] Yang Z Rannala B (1997) Bayesian phylogenetic inference using DNA sequences: A Markov Chain Monte Carlo Method. Molecular Biology and Evolution 14(7): 717–724. 10.1093/oxfordjournals.molbev.a025811 9214744

[B33] Zhang RZ (1992) The Dry Valley of the Hengduan Mountains Regions. Science Press, Beijing, China, 6–20. [In Chinese]

[B34] Zhao EM Zhao KT Zhou KY (1999) Fauna Sinica, Reptilia (Vol. 2): Squamata, Lacertilia. Science Press, Beijing, China, 123–124. [In Chinese]

